# Perceived barriers of heart failure nurses and cardiologists in using clinical decision support systems in the treatment of heart failure patients

**DOI:** 10.1186/1472-6947-13-54

**Published:** 2013-04-26

**Authors:** Arjen E de Vries, Martje HL van der Wal, Maurice MW Nieuwenhuis, Richard M de Jong, Rene B van Dijk, Tiny Jaarsma, Hans L Hillege, Rene J Jorna

**Affiliations:** 1 Department of Cardiology, University Medical Centre Groningen, Hanzeplein 1, PO Box 30.001, Groningen, 9700 RB, The Netherlands; 2 Department of Cardiology, Martini Hospital, Groningen, Groningen, The Netherlands; 3 Department of Social- and Welfare Studies, Linköping University, Faculty of Health Sciences, Norrköping, Sweden; 4 Faculty of Behavioral and Social Sciences, University of Groningen, Groningen, The Netherlands

## Abstract

**Background:**

Clinical Decision Support Systems (CDSSs) can support guideline adherence in heart failure (HF) patients. However, the use of CDSSs is limited and barriers in working with CDSSs have been described as a major obstacle. It is unknown if barriers to CDSSs are present and differ between HF nurses and cardiologists. Therefore the aims of this study are; 1. Explore the type and number of perceived barriers of HF nurses and cardiologists to use a CDSS in the treatment of HF patients. 2. Explore possible differences in perceived barriers between two groups. 3. Assess the relevance and influence of knowledge management (KM) on Responsibility/Trust (R&T) and Barriers/Threats (B&T).

**Methods:**

A questionnaire was developed including; B&T, R&T, and KM. For analyses, descriptive techniques, 2-tailed Pearson correlation tests, and multiple regression analyses were performed.

**Results:**

The response- rate of 220 questionnaires was 74%. Barriers were found for cardiologists and HF nurses in all the constructs. Sixty-five percent did not want to be dependent on a CDSS. Nevertheless thirty-six percent of HF nurses and 50% of cardiologists stated that a CDSS can optimize HF medication. No relationship between constructs and age; gender; years of work experience; general computer experience and email/internet were observed. In the group of HF nurses a positive correlation (r .33, *P*<.01) between years of using the internet and R&T was found. In both groups KM was associated with the constructs B&T (*B*=.55, *P*=<.01) and R&T (*B*=.50, *P*=<.01).

**Conclusions:**

Both cardiologists and HF-nurses perceived barriers in working with a CDSS in all of the examined constructs. KM has a strong positive correlation with perceived barriers, indicating that increasing knowledge about CDSSs can decrease their barriers.

## Background

With a growing elderly population and improved survival after myocardial infarction, the number of patients with heart failure (HF) is increasing. HF is associated with a high re-admission and mortality rate [1]. In order to reduce these rates, many strategies have been developed over the years. The structural application of disease management programs is one such important strategy, and proven to be an important contribution to the reduction of HF related readmissions [2-5]. Disease management programs can be effective in improving the outcomes of HF patients and are therefore advised in recent HF guidelines [5,6]. Since the introduction of those guidelines for both pharmacological and non-pharmacological treatment of HF patients, more patients have been treated with evidence based medication, [7-9] and clinical outcomes of fewer cardiovascular hospitalizations have been observed [10]. However, healthcare providers still experience difficulties when implementing those guidelines in daily practice [11]. The ESC HF pilot survey [12] showed that the rate of prescribed medication that adheres to the guidelines is satisfactory, but the number of patients that receive the optimal dose of ACE-inhibitors, Beta-blockers, and Aldosteron antagonists nevertheless still remains suboptimal. To improve guideline adherence, clinical decision support systems (CDSSs) could, for instance, provide advice and support in prescribing the optimal doses of medication, [13] help with managing the complex care process of HF patients, and improve guideline implementation [14]. There are many definitions of a CDSS [15], but the core principle remains the same throughout. Based on the literature, a CDSS can be said to provide software-based healthcare-related advice to assist doctors and nurses in making decisions and developing solutions, and is often used in complex or non-routine situations.

There is evidence that when using a CDSS, the performance of healthcare providers on clinical outcomes in general improves the quality of care significantly [14,16-18] and it is to be expected that the attitude on CDSS of healthcare providers will influence the actual use and clinical value of using CDSS. However at this moment there is few data available on the attitude of healthcare providers related to patient outcomes. It is known that, despite this evidence for the effectiveness of CDSS, a widespread development, evaluation and implementation of CDSSs, especially in HF clinics, is lacking [19,20]. One of the reasons for this underutilization seems to be a certain level of mistrust or user resistance to CDSSs, [21,22] which has been described as a major barrier for implementing and using CDSSs [22-25]. To suit the needs of users, in order to obtain a successful use of CDSS, the process of designing and the evaluation of a CDSS is described as a critical characteristic [26]. It requires a multidisciplinary process of feedback and software adaption.

### Theoretical background

In general, barriers to guideline adherence consist of a lack of awareness, a lack of agreement or perceived self-efficacy to change, minimal outcome expectancy, and inertia associated with a lack of faith in existing treatment practices [27]. For this study a practical working definition of barriers was defined as: ‘’a barrier is the HF healthcare worker’s perception or estimation of the level of (objectively or subjectively) experienced obstacles”. This indicates that a (perceived) barrier is the result of a complex mental process, in which earlier experiences, beliefs, social environment, and education influence the number of experienced barriers, both in facilitators and in perceived barriers.

Varonen et al. [21] identified potential barriers and facilitators of general physicians to use CDSSs such as earlier experience with dysfunctional computer systems, potential harms to the doctor-patient relationship, unclear responsibilities, threats to clinicians’ autonomy, and extra workload due to excessive reminders. Poor computer skills can also be a barrier to the implementation of a CDSS [28]. The next generation of healthcare providers, however might bring with them a higher level of computer literacy, thus possibly helping the implementation of a CDSS. Knowledge of a CDSS and management of knowledge itself, further described as knowledge management (KM) [29] (i.e., understanding the underlying process of dataflow, the establishment of decisions made by a CDSS, and the assessment of the value of automatically conducted advice by self-generated data input) have been described in earlier research as strong influencers (positive facilitators), for reducing barriers. However, at this moment there is limited data about the type and number of barriers in working with CDSSs, experienced by healthcare providers caring for HF patients. Therefore we decided to perform this study to increase knowledge about barriers to the adaption of CDSSs.

The aims of the present study are:

1: To explore the type and number of perceived barriers of HF nurses and cardiologists in using a CDSS in the treatment of HF patients.

2: To explore possible differences in perceived barriers between two groups of respondents (cardiologists / HF nurses).

3: To assess the relevance and influence of KM on Responsibility/Trust (R&T) and Barriers/Threats (B&T).

## Methods

### Development of the questionnaire

Previously described barriers have been classified in five constructs; trust, responsibilities, threats, resistance, and KM. Since no valid instruments have been developed to measure barriers concerning CDSSs in the domain of HF, a questionnaire based on earlier findings of Varonen [21], Leslie [28], Short [22], and Toth-Pal [23] was developed. In this study, the various items to be used in the five constructs were first defined (Table 1), and later reduced by means of interviews and pilot-testing with pilot responders (10 cardiologists and 20 HF nurses) to a set of item-groups. The final questionnaire consists of 49 items, focusing on perceived barriers using a 5-point Likert type rating scale. (Additioanl file 1: Table S2; questions of perceived barriers on CDSS).

**Table 1:** Definition of constructs

***Construct***	***Definition***
Responsibility	The extent to which the user can take accountability for their (professional) actions and its consequences for the (professional) actions by others.
Trust	The expectation of the user that the offered CDSS is doing what is promised and that you can rely on it.
Barrier	A user objectively or subjectively experienced obstacles to the use of a CDSS
Threat	Feeling of doom combined with an experience of threats or danger that is associated with the offered CDSS.
Knowledge management	The structured, continuous process of developing, sharing, learning, and applying knowledge.

### Validation process of the questionnaire

To test the questionnaire, a group of 30 pilot responders, representing the future research population, completed the questionnaire. The original two constructs, ‘responsibility’ and ‘trust’ could be pooled together to form one scale (R&T). The items belonging to the original constructs ‘threats’ and ‘resistance’, could similarly be grouped together in a single scale, named B&T. The items of the fifth construct formed the scale of KM. Since a lower score on each separate item (1 = totally agree to 5 = totally disagree), indicates more knowledge, a lower score on this construct also indicates more knowledge about a CDSS. After all responders filled in the questionnaire (N=162), the reliability of the questionnaire in terms of Cronbach’s alpha was .85 for the total scale. Cronbach’s alpha for the subscales ranged from .67 to .79. To identify possibly non-observed variables, or new combinations of variables which could indicate another new construct, a factor analysis was performed. However, no new insights for combining items differently were found. The factor analysis thus supported the decision to combine the original five constructs into three (new) main constructs.

### Statistical analyses

Descriptive statistics were used to characterize the study population (mean and SD) and to describe the results of the 49 questions. To examine a possible correlation between characteristics of the respondents and the three constructs, ‘R&T’, ‘B&T’, and ‘KM’, 2-tailed Pearson correlation tests were used. To assess an association of R&T and B&T with KM and the effect of four other theoretically relevant variables (age, years of experiences in current position, years of experience in working with computers, and the use of telemonitoring), multiple regression analyses were performed using the “Forward” selection method [30]. All analyses were conducted for both the whole group and the separate groups (cardiologists, HF nurses). Missing values (3%) in the questionnaires were corrected by replacing them with mean values per construct. Statistical analyses were performed using PASW version 18.0 for Windows.

## Results

### Study population

In March 2011, 220 questionnaires were sent out to all 110 HF clinics in the Netherlands. Because we assume that most HF teams consisted of at least one cardiologist and one HF nurse we estimate that there are approximately 110 HF dedicated cardiologists and 110 HF nurses in the Netherlands. The questionnaire was therefore addressed to the cardiologist and the HF nurse, working as a team, to all HF outpatient clinics. In June 2011 the response period ended, bringing the total response-rate to 74% (total 162 out of 220 questionnaires, 36 questionnaires completed by cardiologists; = 32% of the 110 estimated HF dedicated cardiologists and 126 questionnaires completed by HF nurses = 114% of the 110 estimated HF nurses). The response-rate of the HF nurses is more than 100% because in some cases a colleague nurse in a HF clinic also filled in the questionnaire which was addressed to the cardiologist. In total 35% percent of the respondents had experience in working with telemonitoring systems. This experience mainly consisted of the use of non-invasive devices such as weight scale, blood pressure measurement and electrocardiogram. The generated data were transferred by means of a health monitor, by the internet, to a data center or hospital respectively. If values were outside of the pre-defined ranges, automatically generated alerts and alarms were sent to HF nurses in order to warn them for a possible deterioration of their HF patients.

### Basic characteristics of the study population

Respondents had a mean age of 48 year, and 68% were female. Of the total group respondents, 22% (n=36) were cardiologists and 78% (n=126) were HF nurses. The mean years of work experience in the current position was 14 ± 9 years, and the respondents worked with HF patients for an average of 19 ± 10 hours a week. The mean experience in years of working with computers was 17 ± 6 years. Cardiologists have more experiences in years of working with computers in general (*p* = 0.01) as in more complex computer routine as working with operating systems (*p* = 0.02) and working with software applications (*p* = <0.01). One third of the total respondents had experience in using telemonitoring. Because most of the telemonitoring systems that are used in the Netherlands have some CDSS functionality incorporated (e.g., advice to take action based on incoming alerts) it seems justified to assume that 30% of the respondents have more or less experiences in using CDSS and therefore responded to the questionnaire based on practical experiences. For all other items regarding baseline characteristics we refer to Table 2.

**Table 2:** Summary of baseline characteristics perceived barriers in CDSS (N=162)

	**Cardiologist**		**HF nurse**			**Total**	
***Characteristic***	**(N=36)**	**SD**	**(N=126)**	**SD**	***P*-value**	**(N=162)**	**SD**
Age (mean),y	50	8	47	9	0.93	48	8
Female sex (%)	9(25)		102(82)		<0.01	111(68)	
***Work region***					0.42		
North	9		27			36	
Middle	13		45			58	
South	12		53			65	
***Education***							
University	34		5			39	
Master			40			40	
Applied science			86			86	
Years of experience in current position (mean)	16	9	6	3	<0.01	8	6
Working hours per week with HF patients (mean)	11	10	21	8	<0.01	19	10
***Experience with computers***							
Total in years	19	7	16	5	0.03	17	6
Operating systems	16	7	13	5	0.02	14	6
Software applications	16	6	12	5	0.01	13	6
Programming language							
Email	13	5	13	4	0.47	13	5
Internet	13	4	13	4	0.96	13	5
Use of telemonitoring systems (%)	49		32		0.09	35	

### Responsibility and trust

*Towards more barriers;* sixty-five percent of the respondents indicated they believe that a CDSS can make mistakes. The “clinical expertise” of the healthcare provider was rated as more important and not easily replaced by a computer. The human factor in interpreting clinical patient data and making decisions on treatments was estimated as more important than an advice from a CDSS (98%). Ninety percent stated that advice of a CDSS should always be checked. Seventy-nine percent stated that they are responsible for the treatment of “their” patients and not a CDSS. Forty-nine percent of the respondents stated that they always checked an advice given by a CDSS and 87% stated that they will always check how a CDSS generates an advice.

*Towards less barriers;* most respondents stated that a CDSS can give useful advice about the treatment they should implement (80%). Thirty-five percent reported that in their opinion a CDSS is able to assess patient data, and 18% of the respondents reported that they would easily heed to an advice given by a CDSS.

### Barriers and threats

*Towards more barriers;* nearly 75% of the respondents were uncertain about the time it will take to work with a CDSS during their patient contact. More than 80% of the respondents did not know whether a CDSS especially designed for HF patients would be convenient. Seventy percent stated that they would always notice if deviations or shortcomings in data, such as in laboratory tests, physical examinations, and medication appear or are present. Seventy percent were not sure or disagreed that following a treatment advice given by a CDSS has no influence on whether or not the patient takes a doctor or a nurse seriously. Thirty-nine percent reported that a “normal/standard” patient record provides sufficient information. Ninety percent disagreed with the statement that anyone can treat a HF patient with the help of a CDSS.

*Towards less barriers;* sixty-two percent of the respondents reported that advice of a CDSS on how to treat a HF patient is a welcome supplement to their own expertise, whereas another 30% reported that a CDSS that works with guidelines can be adapted quickly. A total of 46% of the respondents stated that the use of a CDSS will not influence the relationship with their patients and 55% stated that a CDSS supplements their independency as a HF care expert.

### Knowledge management

*Towards less barriers;* sixty percent of the respondents declared that a CDSS can give advice about treatment and gives insight in the treatment process of a HF patient and thus has additional value for the treatment. Eighty percent proclaimed that information supplied by a CDSS adds (additional) value to their own knowledge of treating HF patients. The respondents stated that their ability to apply guidelines improved and they felt positive about a warning or alert given by a CDSS about the course of the treatment. Forty percent reported that with the help of a CDSS they are better able to adjust optimal dosages of medication.

*Towards more barriers;* Twenty percent stated that they are not better able to adjust optimal dosages of medication with the help of a CDSS. Another 10% stated that a CDSS that provides advice about treating heart failure gives no insight into the treatment process. For all questions: see Additional file 1: Table S1).

### Differences between cardiologists and HF nurses

Differences were found between the groups of respondents (Additional file 1: Table S1). In the 2-tailed Pearson correlation tests of the total group, we found no significant correlation between age, gender, years of work experience, general computer experience or experience with operating systems, computer programs, and email/internet within the three constructs (B&T, R&T and KM). The most prominent differences with respect to the three constructs are described in Table 3. In the subgroup of HF nurses there was a significant positive correlation between years of using the internet *(r=* .33, P<0.01), years of using email *(r=* .23, P<0.05), and years of computer experience *(r=* .29, *P*<0.01) in relation to R&T. There was also a positive correlation between years of computer experience and the construct KM *(r=* .22, *P*<0.05). In the multiple regression analyses (Table 4), different variables and the construct of KM itself were tested on their possible association with B&T and R&T These variables were chosen for their relevance and strong presence in the baseline characteristics. Only KM had a strong, independent association with the constructs B&T (*B*= .55, P=<.01) and R&T (*B*=.50, *P*=<.01). Respondents who reported that they were currently using telemonitoring systems tended to experience less B&T (*B*= −.13, *P*=.06).

**Table 3:** Differences found in response to a selection of the questions between HF nurses (HF) and cardiologists (cardio)

	**% agree**		**diff.**	***P*-value**	**% not agree**		**diff.**	***P*-value**
	**Cardio**	**HF**			**Cardio**	**HF**		
	**Knowledge management**							
A CDSS gives me useful information about the treatment.	43	68	26	0.02	3	0	3	
A warning from a CDSS about the course of treatment is very welcome.	62	36	26	0.74	6	21	15	
I can determine the optimal dose of heart failure medication much faster with the help of a CDSS.	50	36	14	0.26	12	21	9	
	**Responsibility and Trust (R&T)**							
The treatment I prescribe to my patients could depend on a CDSS.	65	36	29	0.49	27	23	4	
A CDSS can give advice about the treatment I should implement.	67	81	14	0.37	6	6	0.2	
The Healthcare Inspectorate should stimulate the use of a CDSS that can provide treatment advice.	18	21	4		32	18	14	0.03
	**Barriers and Threats (B&T)**							
When I use a computer during patient contacts, this does not influence my relationship with the patient.	32	45	13		55	29	26	0.05
A CDSS could play a dominant role during a consultation.	9	20	11		53	38	15	0.08
A CDSS reduces my work load.	0	10	10		49	37	12	0.21
The application of guidelines by a CDSS is still in its infancy.	64	36	28	<0.01	0	2	2	
A CDSS that works with guidelines can be adapted quickly.	44	27	17	0.97	10	2	8	

**Table 4:** Multi variate regression analyses; association of B&T and R&T with independent variables (all respondents)

**Barriers and Threats (B&T)**				
	**[95% CI]**			
	**B (SE)**	**Lower**	**Upper**	**P-value**
Age	-.02 (.05)	-.10	.07	.74
Knowledge management	.55 (.09)	.57	.92	<.01
Years of experience in current function	.09 (.06)	-.05	.19	.24
Years of experience in working with computers	-.03 (.06)	-.14	.09	.63
Use of telemonitoring	-.13 (.05)	−2.08	.06	.06
**Responsibility and Trust (R&T)**				
	**[95% CI]**			
	**B (SE)**	**Lower**	**Upper**	**P-value**
Age	-.01 (.04)	-.09	.08	.91
Knowledge management	.50 (.09)	.46	.82	<.01
Years of experience in current function	.11 (.06)	-.03	.20	.16
Years of experience in working with computers	-.03 (.06)	-.09	.14	.65
Use of telemonitoring	-.09 (.53)	−1.70	.40	.23

## Discussion

In this first study to examine the number of perceived barriers on working with CDSSs by cardiologists and nurses, we found a substantial number of perceived barriers in using CDSS in two of the three constructs (R&T and B&T). The results of the construct KM in general showed that most respondents do see the added value of a CDSS in terms of learning, being better informed about the treatment, and a possibly better guideline adherence. Our study explored whether demographic factors, education, and/or computer experience are related to the number of perceived barriers in using a CDSS. Understanding these barriers is important because the implementation of CDSSs in HF care can play a significant role in optimizing the management of HF medication according to the guidelines and it is generally known that optimizing medication management in HF patients reduces readmission and mortality rates.

The respondents of this study were both highly experienced in working with HF patients, and in working with computers, email, the internet, and software programs. However, contrary to results reported in earlier studies, [21,28] in the subgroup of cardiologists no correlation was shown between age; gender; experiences in working with computers, programs, and software on perceived barriers. The often heard presumption that “working with computers” positively influences the capability to work with CDSSs and hence causes fewer barriers was therefore not proven for the group of cardiologists in this study. However, in the subgroup of HF nurses different types of experience in working with computers strongly influenced the number of perceived barriers, in particular with respect to responsibility and trust (R&T).

More experience in working with computers was related to higher scores on R&T and therefore to a lower number of perceived barriers. This dissimilarity between cardiologists and HF nurses might be explained by differences in professional position and the amount of autonomy and/or final responsibility in treatment decisions. It is imaginable that HF nurses experience more support from a CDSS as a ‘helper’ in making important treatment decisions instead of experiencing a loss of autonomy.

A high percentage of respondents who already worked with telemonitoring were found. This could have influenced the number of perceived barriers. Beside these experiences in working with CDSS’s, working with this new technology probably indicates a certain preference for technology. For this reason we corrected by using telemonitoring as a covariate in the multivariate regression analyses. However, no significant relation between the constructs and the use of telemonitoring itself was found. It is remarkable that when a CDSS is less informative and instead gives more direct and stringent advice (alerts, warnings, and request for additional information), respondents seem to have more hesitations or reserves towards a CDSS. This is interesting because this functionality in particular marks the main difference between ‘regular’ software and a CDSS. A possible explanation for this finding could be that this specific functionality of a CDSS is seen as causing a loss of professional autonomy. In general, the barriers to the adaptation of a CDSS are similar in both the groups of HF nurses and the group of cardiologists, although some differences were found between the two groups. It is difficult to interpretate these differences because the scores on the constructs towards a greater or lesser number of barriers fluctuated as much in the group of HF nurses as in the group of cardiologists.

However, warnings, alerts, and advice given by a CDSS to enable better guideline adherence seem to be less adopted by the group of HF nurses. This could be a result of who is actually performing the HF care in daily practice.

There were some known prejudices about CDSSs found in this study. We have seen statements confirmed or reject such as ‘CDSS is still in its infancy’, ‘CDSS can reduce my workload’, and ‘CDSS can play a dominant role during a consult’. These prejudices or presumptions can be seen as barriers and are associated with a lower adoption level of a CDSS, and can therefore possibly result in a decrease of adherence to guidelines. The construct KM itself was, as expected, not a barrier and was associated with the constructs B&T and R&T. This indicates that a higher level of knowledge in understanding the underlying mechanism of a CDSS leads to a decrease in barriers. This is understandable, because comprehension of how a CDSS works gives a more realistic view of the possibilities and impossibilities of a CDSS. The fact that a CDSS is not a magic black box, but will only generate advice by means of predefined formulas and data provided by the HF professionals themselves, will give more attention to the system’s capabilities. This could prevent disillusions and lead to a more positive attitude towards CDSSs. In order to use a CDSS successfully, the process of developing and evaluation is as even important as decreasing barriers in using it. Earlier research showed critical components that are of decisive order in a optimal use; e.g., determining the scope of the CDSS, involving target users in the development, and the authority of the CDSS. Finally, we stress the importance of further research on the attitude of healthcare providers in using a CDSS regarding to patient-related outcomes [26].

### Limitations

This study has some potential limitations. First of all, although the overall response rate of this questionnaire was more than reasonable (74%), the actual response of the subgroup of cardiologists was only 32% (36 out of 110 expected questionnaires), making the sample of cardiologists rather small with consequently effect for the power of this study. Second, the following two questions were central when developing the questionnaire: ‘Did we identify the right constructs and independent variables to measure the strength of perceived barriers’, and ‘Are the constructs representative enough to determine the perceived barriers’? In our pilot we found that four of the five defined constructs (responsibility with trust, and barriers with threats), are strongly aligned to each other and exist in a continuum as it were. Because of this continuum, it was difficult to measure these constructs separately. Combining these related constructs therefore seemed a logical and explainable action. The identified constructs used in this research were based on the available literature on barriers to the adoption of CDSSs. Therefore, we believe that we have sufficient reasons to acknowledge that the identified constructs indeed give information on barriers to using CDSS, although further research should be conducted to give more insights in this specific field. Because this survey was only sent to cardiologists and HF nurses, working in the field of HF care, other workers in this field; e.g., family practitioners, general cardiac nurses and community based nurses were not included. This affects the generizability of the study. Finally, we are aware of the disadvantages of using data based on self-reports. Unfortunately, in the Netherlands we do not have a long history of experience in working with CDSSs in the field of HF.

## Conclusions

HF nurses and cardiologists working in HF clinics in the Netherlands - while taking into account differences between the groups - have substantial perceived barriers in all three examined constructs when working with a CDSS. Characteristics such as age, gender, and experience in working with computers did not influence the strength of perceived barriers in the group of cardiologists. However, in the group of HF nurses, experience in working with computers and with email and the internet, had a strong effect on B&T and R&T. These are therefore factors that should be taken into consideration, as described in earlier studies. KM has a strong, significant association with perceived barriers, indicating that users who find the CDSS useful experienced less percieved barriers and that suggests that increasing knowledge will decrease barriers. In spite of the presumption that telemonitoring devices are ‘smart devices’ and require a higher level of computer literacy, no significant association between the use of telemonitoring and a decrease in barriers to the use of CDSSs was found.

## Competing interests

The authors of the study declare no competing interests.

## Authors’ contributions

AEdV: designing, conducting, analyzing and interpretation of data and drafting the article. RJ and HH (Epidemiologist, general manager of the Trial Coordination Centre) for their supervision of the statistical methods and data analyzing. MN, PhD for his help with conducting and analyzing the data. All other authors made substantial contributions to the article and revising it critically for important intellectual content. All authors read and approved the final manuscript.

## Pre-publication history

The pre-publication history for this paper can be accessed here:

http://www.biomedcentral.com/1472-6947/13/54/prepub

## Supplementary Material

Additional file 1: Table S1 49-items of the Perceived Barriers on CDSS questionnaire and scores in mean, SD and percentage agree, disagree and neutral of cardiologist, HF nurses and all respondents. Constructs; R&T (responsibility and trust) B&T (barriers and threats) KM (knowledge management). Click here for file
