# Supplementary material for: Perceived barriers of heart failure nurses and cardiologists in using clinical decision support systems in the treatment of heart failure patients
Source: BMC Med Inform Decis Mak. 2013 Apr 26;13:54. doi: 10.1186/1472-6947-13-54 (PMC3651365; doi:10.1186/1472-6947-13-54)
Supplement: Additional file 1: Table S1 — 49-items of the Perceived Barriers on CDSS questionnaire and scores in mean, SD and percentage agree, disagree and neutral of cardiologist, HF nurses and all respondents. Constructs; R&T (responsibility and trust) B&T (barriers and threats) KM (knowledge management). [file 1472-6947-13-54-S1.doc]

Additional file 1: Table S2. 49-items of the Perceived Barriers on CDSS questionnaire and scores in mean, SD and percentage agree, disagree and neutral of cardiologist, HF nurses and all respondents. Constructs; R&T (responsibility and trust) B&T (barriers and threats) KM (knowledge management).

|  |  |  | **Cardiologists N=36 (A)** | **HF nurses N=126 (B)** | **A-B** | **All respondents N=162** |  |  |  |  |  |  |  |  |  |
| --- | --- | --- | --- | --- | --- | --- | --- | --- | --- | --- | --- | --- | --- | --- | --- |
|  | **Questions of the perceived barriers on CDSS questionnaire** | **Construct** | **Mean±SD** | **%agree** | **%neutral** | **%disagree** | **Mean±SD** | **%agree** | **%neutral** | **%disagree** | ***P*-value** | **Mean±SD** | **%agree** | **%neutral** | **%disagree** |
| 1 | A CDSS that gave me advice about how to treat heart failure would be a great help. | R&T | 2.6± .9 | 49 | 34 | 14 | 2.7±.8 | 35 | 46 | 15 | 0.38 | 2.7±.8 | 41 | 44 | 15 |
| 2 | Computers cannot make mistakes. | R&T | 3.5±1 | 23 | 17 | 60 | 3.6±.9 | 15 | 18 | 66 | 0.32 | 3.6±1 | 17 | 18 | 65 |
| 3 | When I use a computer during patient contacts, this does not influence my relationship with the patient. | B&T | 3.3±1 | 31 | 14 | 54 | 2.9±.9 | 42 | 22 | 28 | **0.05** | 2.9±1 | 46 | 22 | 36 |
| 4 | My clinical expertise could be replaced by a computer. | R&T | 4.6±.5 | 0 | 0 | 100 | 4.4±.6 | 2 | 2 | 96 | 0.28 | 4.5±.6 | 13 | 1 | 97 |
| 5 | When I follow the treatment advice given by a CDSS, my patient takes me just as seriously as always. | B&T | 2.9±.7 | 29 | 53 | 18 | 2.9±.8 | 30 | 46 | 22 | 0.87 | 2.9±.8 | 31 | 49 | 20 |
| 6 | It is not necessary to check the advice given by a CDSS. | R&T | 4.1±.9 | 9 | 3 | 89 | 4.1±.6 | 2 | 6 | 90 | 0.74 | 4.1±.7 | 4 | 6 | 90 |
| 7 | The advice given by a CDSS is easy to follow. | R&T | 2.9±.5 | 18 | 76 | 6 | 2.9±.5 | 19 | 69 | 12 | 0.83 | 2.9±.5 | 18 | 72 | 10 |
| 8 | I readily adopt the advice given by a CDSS. | R&T | 3.2±.7 | 15 | 59 | 26 | 3.2±.5 | 5 | 66 | 29 | 0.48 | 3.2±.6 | 7 | 65 | 28 |
| 9 | A CDSS that supports me does not undermine my independence as a health care provider. | B&T | 2.6±.9 | 56 | 23 | 20 | 2.5±.5 | 58 | 27 | 13 | 0.43 | 2.5±.8 | 60 | 26 | 14 |
| 10 | The treatment I prescribe to my patients could depend on a CDSS. | R&T | 2.8±.9 | 64 | 29 | 26 | 2.9±.7 | 36 | 40 | 22 | 0.49 | 2.9±.8 | 36 | 39 | 25 |
| 11 | A CDSS could play a dominant role during a consultation. | B&T | 3.5±.7 | 9 | 38 | 53 | 3.2±.8 | 20 | 42 | 38 | 0.08 | 3.3±.8 | 17 | 42 | 41 |
| 12 | A CDSS reduces my work load. | B&T | 3.5±.6 | 0 | 51 | 48 | 3.3±.7 | 10 | 53 | 37 | 0.21 | 3.4±.7 | 7 | 54 | 39 |
| 13 | A CDSS that provides advice about treating heart failure gives me insight into my treatment process. | KM | 2.6±.7 | 53 | 34 | 12 | 2.5±.6 | 56 | 35 | 8 | 0.67 | 2.5±.7 | 57 | 35 | 9 |
| 14 | A CDSS that can advise me about how to treat my patients is a welcome supplement. | B&T | 2.3±.4 | 65 | 35 | 0 | 2.4±.6 | 62 | 33 | 5 | 0.59 | 2.4±.6 | 63 | 33 | 4 |
| 15 | As a health care provider, I am responsible for the treatment I provide. | R&T | 4.6±.4 | 100 | 0 | 0 | 4.5±.5 | 97 | 2 | 1 | 0.30 | 4.5±.6 | 97 | 2 | 1 |
| 16 | A CDSS specifically for heart failure would have added value for the way I do my job. | KM | 2.5±.5 | 45 | 54 | 0 | 2.5±.5 | 54 | 43 | 3 | 0.68 | 2.5±.6 | 52 | 47 | 2 |
| 17 | A CDSS that gives treatment advice only complicates the treatment process. | B&T | 2.8±.6 | 12 | 54 | 33 | 2.7±.6 | 20 | 57 | 22 | 0.10 | 2.8±.6 | 32 | 57 | 11 |
| 18 | The Healthcare Inspectorate should stimulate a CDSS that can provide treatment advice. | R&T | 3.3±.9 | 18 | 50 | 32 | 2.9±.6 | 21 | 61 | 18 | **0.03** | 3.0±.7 | 20 | 59 | 21 |
| 19 | I do not want to be dependent on the manufacturers of a CDSS that gives me advice on how to treat my patients. | R&T | 3.7±.7 | 67 | 24 | 9 | 3.6±.7 | 64 | 29 | 7.2 | 0.97 | 3.6±.8 | 65 | 28 | 8 |
| 20 | I am able to feel responsible for advice given by a CDSS. | R&T | 2.7±.8 | 46 | 29 | 23 | 2.6±.5 | 51 | 35 | 13 | 0.58 | 2.6±.8 | 51 | 34 | 15 |
| 21 | During consultations, the patient file provides ample information. | B&T | 3.0±.9 | 32 | 38 | 29 | 3.1±.8 | 41 | 28 | 31 | 0.75 | 3.1±.9 | 39 | 31 | 31 |
| 22 | A CDSS can give advice about the treatment I should implement. | R&T | 2.3±.7 | 67 | 26 | 6 | 2.4±.5 | 81 | 14 | 6 | 0.37 | 2.3±.6 | 78 | 17 | 6 |
| 23 | I always have to assess the advice of a CDSS. | R&T | 4.3±.8 | 89 | 9 | 3 | 4.2±.5 | 95 | 3 | 2 | 0.65 | 4.2±.6 | 94 | 4 | 2 |
| 24 | A CDSS gives me useful information about the treatment. | KM | 2.5±.7 | 42 | 54 | 3 | 2.3±.4 | 68 | 32 | 0 | **0.02** | 2.4±.6 | 62 | 37 | 1 |
| 25 | A CDSS that works with guidelines can be adapted quickly. | B&T | 2.7±.7 | 44 | 47 | 9 | 2.7±.5 | 27 | 71 | 2 | 0.97 | 2.7±.6 | 30 | 66 | 3 |
| 26 | Information from a CDSS about treatment can supplement my own knowledge. | KM | 2.2±.6 | 83 | 14 | 3 | 2.2±.5 | 81 | 15 | 3 | 0.86 | 2.2±.5 | 81 | 16 | 3 |
| 27 | A CDSS makes it easy to keep heart failure guidelines and protocols up to date. | KM | 2.5±.7 | 57 | 34 | 9 | 2.4±.5 | 58 | 41 | 1 | 0.18 | 2.4±.6 | 58 | 40 | 2 |
| 28 | A CDSS is able to assess patient data. | R&T | 2.9±.8 | 39 | 45 | 15 | 2.8±.7 | 35 | 49 | 16 | 0.74 | 2.8±.7 | 36 | 48 | 16 |
| 29 | A CDSS can indicate treatment priorities. | R&T | 2.7±.8 | 50 | 29 | 20 | 2.7±.7 | 43 | 47 | 10 | 0.62 | 2.7±.7 | 44 | 44 | 12 |
| 30 | Adapting the advice of a CDSS costs me extra time. | B&T | 3.4±.8 | 41 | 53 | 6 | 3.3±.6 | 32 | 62 | 6 | 0.40 | 3.3±.6 | 33 | 61 | 6 |
| 31 | I can learn from a CDSS. | KM | 2.4±.6 | 65 | 29 | 6 | 2.3±.5 | 65 | 34 | 1 | 0.38 | 2.4±.6 | 65 | 33 | 2 |
| 32 | Patient care improves with a CDSS. | B&T | 2.9±.7 | 26 | 66 | 9 | 2.7±.5 | 32 | 64 | 4 | 0.15 | 2.8±.6 | 30 | 65 | 5 |
| 33 | A CDSS can help me apply guidelines. | KM | 2.3±.6 | 67 | 29 | 3 | 2.3±.5 | 69 | 31 | 0 | 1.00 | 2.3±.5 | 63 | 31 | 1 |
| 34 | A CDSS supplements my independence as a heart failure care expert. | B&T | 2.7±.8 | 47 | 38 | 15 | 2.4±.6 | 60 | 34 | 6 | 0.07 | 2.5±.7 | 55 | 36 | 8 |
| 35 | A CDSS that gives advice about treatment is dependent on other systems,( laboratories and/or electronic patient files) | KM | 3.6±.6 | 62 | 27 | 6 | 3.9±.6 | 74 | 25 | 1 | **0.04** | 3.8±.7 | 71 | 27 | 2 |
| 36 | Anyone can treat a heart failure patient with the help of a CDSS. | B&T | 1.7±.7 | 3 | 9 | 89 | 1.6±.6 | 0 | 80 | 92 | 0.35 | 1.6±.7 | 1 | 9 | 91 |
| 37 | Using a CDSS during a patient contact takes too much time. | B&T | 3.1±.5 | 18 | 73 | 9 | 3.0±.5 | 11 | 76 | 13 | 0.18 | 3.0±.5 | 13 | 75 | 12 |
| 38 | A CDSS that gives advice is confusing. | B&T | 3.0±.8 | 29 | 38 | 32 | 2.9±.7 | 17 | 54 | 29 | 0.59 | 2.9±.7 | 20 | 51 | 29 |
| 39 | Advice from a CDSS must always be adapted. | B&T | 2.9±.6 | 12 | 64 | 24 | 3.0±.5 | 13 | 73 | 14 | 0.43 | 3.0±.6 | 13 | 71 | 16 |
| 40 | If guidelines are included in a CDSS, I will always be up to date. | R&T | 2.7±.9 | 54 | 29 | 17 | 2.6±.6 | 50 | 43 | 7 | 0.40 | 2.6±.7 | 51 | 40 | 9 |
| 41 | A CDSS specifically designed for heart failure is easy to use. | B&T | 3.0±.5 | 15 | 73 | 12 | 2.9±.4 | 11 | 84 | 4 | 0.39 | 2.9±.4 | 12 | 82 | 6 |
| 42 | A warning from a CDSS about the course of treatment is very welcome. | KM | 2.4±.7 | 62 | 32 | 6 | 2.4±.6 | 36 | 43 | 21 | 0.74 | 2.4±.6 | 62 | 34 | 4 |
| 43 | I can determine the optimal dose of heart failure medication much faster with the help of a CDSS. | KM | 2.7±.7 | 50 | 38 | 12 | 2.8±.8 | 36 | 43 | 21 | 0.26 | 2.8±.8 | 39 | 43 | 19 |
| 44 | A CDSS can provide me with supplementary treatment information. | KM | 2.3±.7 | 76 | 18 | 6 | 2.2±.5 | 80 | 17 | 2 | 0.44 | 2.2±.5 | 79 | 18 | 3 |
| 45 | I must always be able to check how a CDSS arrives at the treatment advice. | R&T | 4.2±.5 | 91 | 9 | 0 | 3.9±.5 | 85 | 13 | 2 | **<0.01** | 4.0±.6 | 86 | 12 | 1 |
| 46 | I always notice abnormal diagnostic values (e.g. kidney function, blood pressure, heart rate) during treatment. | B&T | 3.5±.9 | 60 | 23 | 17 | 3.7±.7 | 71 | 23 | 6 | 0.09 | 3.7±.8 | 68 | 23 | 8 |
| 47 | The application of guidelines by a CDSS is still in its infancy. | B&T | 4.0±.8 | 64 | 36 | 0 | 3.4±.6 | 35 | 62 | 2 | **<0.01** | 3.5±.7 | 41 | 57 | 2 |
| 48 | You need additional computer skills to use a CDSS. | B&T | 3.0±.8 | 29 | 47 | 23 | 3.0±.7 | 23 | 54 | 24 | 0.61 | 3.0±.7 | 24 | 52 | 23 |
| 49 | Other care providers involved with my patient would have to work in the same system as me. | KM | 2.3±.7 | 62 | 32 | 6 | 2.1±.7 | 69 | 30 | 1 | 0.22 | 2.2±.7 | 67 | 31 | 2 |
